# Inflammatory Bowel Disease and Thrombosis: A National Inpatient Sample Study

**DOI:** 10.1055/s-0040-1710506

**Published:** 2020-05-18

**Authors:** Jessica B. Cohen, Diane M. Comer, Jonathan G. Yabes, Margaret V. Ragni

**Affiliations:** 1 Department of Medicine, Division of Hematology/Oncology, University of Pittsburgh Medical Center and Hemophilia Center of Western Pennsylvania, Pittsburgh, Pennsylvania, United States; 2 University of Pittsburgh, Pittsburgh, Pennsylvania, United States; 3 University of Pittsburgh Center for Research on Health Care Data Center, Pittsburgh, Pennsylvania, United States

**Keywords:** inflammatory bowel disease, malignancy, malnutrition, steroids, thrombosis

## Abstract

**Introduction** Thrombosis is more common in inflammatory bowel disease (IBD) patients than the general population, but disease-specific correlates of thrombosis remain unclear.

**Methods** We performed a retrospective analysis of discharge data from the National Inpatient Sample between 2009 and 2014, using International Disease Classification codes to identify IBD and non-IBD patients with or without thrombosis. We used NIS-provided discharge-level weights to reflect prevalence estimates. Categoric variables were analyzed by Rao-Scott Chi-square test, continuous variables by weighted simple linear regression, and covariates associated with thrombosis by weighted multivariable logistic regression.

**Results** Thrombosis prevalence in IBD was significantly greater than in non-IBD, 7.52 versus 4.54%, *p* < 0.0001. IBD patients with thrombosis were older and more likely to be Caucasian than IBD without thrombosis, each *p* < 0.001. Thrombosis occurred most commonly in the mesenteric vein. Thrombotic risk factors in IBD include surgery, ports, malignancy, dehydration, malnutrition, and steroids at 53.7, 13.2, 13.1, 12.4, 8.9, and 8.2%, respectively. Those with thrombosis had greater severity of illness, 1.42 versus 0.96; length of stay, 7.7 versus 5.5 days; and mortality, 3.8 versus 1.5%; all *p* < 0.0001. Adjusting for age and comorbidity, odds ratios for predictors of thrombosis included ports, steroids, malnutrition, and malignancy at 1.73, 1.61, 1.34, and 1.13, respectively, while Asian race, 0.61, was protective, each *p* < 0.001.

**Conclusion** Thrombosis prevalence is 1.7-fold greater in IBD than non-IBD patients. Adjusting for age and comorbidity, the odds ratio for thrombosis in IBD was 73% higher with ports, 61% higher with steroids, 34% with malnutrition, and 13% with malignancy. Whether long-term anticoagulation would benefit the latter is unknown.

## Introduction

Inflammatory bowel disease is a chronic inflammatory disorder of the gastrointestinal (GI) mucosa including Crohn's disease (CD) and ulcerative colitis (UC). An estimated 1.4 million people in the United States currently suffer from IBD, and the incidence is increasing with time. 1 2 3 These disorders are characterized by defective immune regulation in which T-cells in the GI mucosa secrete inflammatory cytokines. 4 5 The subsequent inflammatory response, both acute and chronic, induces a prothrombotic state which is mediated by an increase in procoagulant factors and a decrease in natural anticoagulants and in fibrinolytic activity. 6 More specifically, there is an upregulation of tissue factor and an increase in the platelet count that promotes thrombosis; at the same time, decreased expression of tissue plasminogen activator and increased levels of plasminogen activator inhibitor-1 contribute to decreased fibrinolysis. 7 8 Under these conditions, development of venous thromboembolism (VTE) is one of the most common extrasystemic manifestations of IBD, with a risk 2 to 3 times greater than in the general population. Moderate and severe disease flares, hospitalization, and surgery appear to increase thrombosis risk further. 9 10 11

Current guidelines suggest the use of pharmacologic VTE prophylaxis in IBD patients admitted to the hospital for any reason, especially if the admission is for a disease flare, even if accompanied by nonsevere GI bleeding, as the latter is not a contraindication to anticoagulation. 12 However, surveys of both American and Canadian gastroenterologists reveal a lack of institutional standards, with a wide variation in the application of these guidelines, many of which are based on low-quality evidence. 13 14 Morbidity and mortality remain elevated for these patients, and it is unclear what additional risk factors may place patients at risk for thrombosis. We analyzed the National Inpatient Sample to further characterize the prevalence and risk factors for thrombosis in hospitalized patients with IBD.

## Methods

### Study Design and Data Source

This was a retrospective analysis of discharge data from the National Inpatient Sample (NIS) between January 1, 2009 and December 31, 2014. The NIS is an all-payer database that approximates a 20% stratified sample of discharges from U.S. community hospitals participating in the Healthcare Cost and Utilization Project. 15 It contains unweighted data from more than 7 million yearly hospital stays, and once weighted, estimates approximately 35 million stays. It includes deidentified clinical and nonclinical elements such as primary and secondary diagnoses, patient demographics, payment source, length of stay, and severity and comorbidity measures. Data during this time period were classified by the International Classification of Diseases, Ninth Revision (ICD-9) diagnostic codes.

### Populations

Patient discharges, both with and without IBD and with and without thrombosis, were identified using ICD-9 diagnostic codes 555.0 (regional enteritis) and 556.0 (ulcerative colitis). Codes used to identify venous thrombosis included deep venous thrombosis (451.11, 451.19, 451.2, 451.81, 451.83, 451.9, 453.40, 453.41, 453.42, 453.8, and V12.51), pulmonary embolism (415.11, 415.13, and 415.19), portal vein thrombosis (452.0), splenic vein thrombosis (444.89), mesenteric vein thrombosis (557.0 and 557.1), hepatic vein thrombosis (453.0), and intracranial venous sinus thrombosis (325.0). Arterial thrombosis was identified using the following codes: arterial thrombosis/embolism (444), cerebral artery embolism (434.1), cerebral artery thrombosis (434), and retinal artery occlusion (362.3).

### Potential Risk Factors

The frequency of potential risk factors for thrombosis in patients with IBD was evaluated and identified using diagnosis codes: dehydration (276.5, 639.5, and 998.0), malnutrition (263.0, 263.1, 263.8, and 263.9), ports/central venous catheters (996.1 and 38.93), long-term steroid use (V58.65), postoperative state (V45, 998.9), malignancy (140–239), pregnancy (650), hormone therapy (V25.01, V25.09, and V25.8), immobilization (V49.89), trauma (800–959), and surgical procedures (general procedures: 01–86; abdominal: 45, 46, and 48; and orthopaedic: 76–84).

### Comorbidities

General comorbidities were evaluated using the following codes: diabetes (250–250.3 and 250.7), obesity (278.0), smoking (305.1 and V15.82), hypertension (401–405), hyperlipidemia (272.0, 272.2, and 272.4), cardiac disease (410–414), renal failure (403, 404, 585, and 586), liver disease (571.8, 571.9, and 572.8), cirrhosis (571.5), hepatitis C (070.41, 070.44, 070.51, 070.54, 070.70, and 070.71), HIV (042, 079.53, 795.71, and V08), and transfusion (blood or blood products: V582, 5187, 9647, 9996, 9997, and E8760; platelets: 99.05; and coagulation factors: 99.06). Deyo's modification of the Charlson's comorbidity index was used to assess the severity of illness between groups; by assigning weights to 16 diseases based on the strength of their association with mortality, the index controls for confounding.

### Statistical Analysis

Thrombosis prevalence was estimated among admissions with and without IBD. The two groups were compared with respect to patient characteristics (age, race, and gender), insurance type, length of stay, inpatient mortality, and risk factors for thrombosis. These variables were further compared with and without thrombosis within each IBD group. Univariate analyses for between-group comparisons used Rao-Scott Chi-square test for categorical variables (e.g., gender and risk factors) and weighted simple linear regression for continuous variables (e.g., age). Weighted multivariable logistic regression was performed to identify factors associated with thrombosis among those with IBD, controlling for age and comorbidity. Odds ratios (ORs) and 95% confidence intervals (CIs) were calculated. Multivariable logistic regression was performed to identify factors associated with thrombosis among those with IBD. The multivariable model included covariates selected based on clinical and statistical significance. All analyses used discharge-level weights provided by NIS to reflect national estimates. All analyses were conducted in SAS (SAS version 9.4, SAS corporation), and *p* -values < 0.05 were considered statistically significant.

## Results

### Admission Characteristics

During the 6-year period from January 1, 2009 to December 31, 2014, an unweighted total of 45,123,086 admissions was collected of which 374,315 included a diagnosis code for IBD. The prevalence of thrombosis in patients with IBD (7.51%, *n* = 28,155) was 1.7 times higher than the prevalence of thrombosis in patients without IBD (4.53%, *n* = 2,029,915), *p* < 0.0001. IBD patients had an increased prevalence of thrombotic risk factors including general surgery, abdominal surgery, dehydration, ports/central venous catheters, malnutrition, and long-term steroid use, all *p* < 0.0001, as compared with non-IBD patients ( Table 1 ). Markers of overall health, including the Charlson's score (9.99 vs. 1.25, *p* < 0.0001) and length of stay (5.63 vs. 4.58 days) were significantly worse, while inpatient mortality (1.66 vs. 1.90%, *p* < 0.0001) was significantly lower in IBD patients compared with patients without IBD ( Table 1 ).

**Table 1:** Characteristics of IBD and non-IBD admissions from 2009 to 2014

Baseline characteristics	IBD	No IBD	*p* -Value
	Percent of mean (SE)		
**No. of hospital admissions**			
Actual	**374,315**	**44,748,771**	
Weighted	1,860,564	222,614,880	
**Patient characteristics**			
Age (y)	50.97 (0.03)	48.66 (0.00)	<0.0001
**Race**			<0.0001
Caucasian	79.82	65.76	
African American	10.67	14.98	
Asian	1.03	2.69	
Other	8.48	16.57	
**Gender**			<0.0001
Female	56.86	57.72	
Male	43.14	42.28	
**Thrombosis**			
Any thrombosis	7.51	4.53	<0.0001
Mesenteric vein thrombosis	0.56	0.18	<0.0001
Pulmonary embolism	0.49	0.48	0.4276
Deep venous thrombosis	0.47	0.36	<0.0001
Portal vein thrombosis	0.19	0.06	<0.0001
Cerebral artery embolism	0.16	0.25	<0.0001
Other/splenic vein thrombosis	0.04	0.02	<0.0001
Cerebral artery thrombosis	0.03	0.04	0.0144
Intracranial venous sinus thrombosis	0.03	0.01	<0.0001
Retinal occlusion (artery or vein)	0.03	0.04	0.0011
Hepatic vein thrombosis (Budd–Chiari syndrome)	0.02	0.01	<0.0001
**Risk factors**			
General surgical procedures	52.01	50.23	<0.0001
Abdominal surgery	26.21	4.89	<0.0001
Dehydration	13.15	6.31	<0.0001
Malignancy	9.48	10.28	<0.0001
Ports/central venous catheters	8.07	4.10	<0.0001
Malnutrition	6.03	2.46	<0.0001
Long-term steroid use	5.71	0.99	<0.0001
Orthopedic surgery	4.94	8.33	<0.0001
Trauma	4.15	6.95	<0.0001
Charlson's score comorbidity index	9.99 (0.00)	1.25 (0.00)	<0.0001
Length of stay	5.63 (0.01)	4.58 (0.00)	<0.0001
Discharge status (inpatient mortality)	1.66	1.90	<0.0001

Abbreviations: IBD, inflammatory bowel disease; SE, standard error.

Among patients with IBD, those with thrombosis were significantly older (57.48 vs. 50.44 years, *p* < 0.0001), more likely to be Caucasian (81.54 vs. 79.68%, *p* < 0.0001), and less likely to be Asian (0.62 vs. 1.06%, *p* < 0.0001), than those without thrombosis. There was no significant gender effect on thrombosis prevalence in IBD patients ( Table 2 ).

**Table 2:** Patient characteristics and risk factors for thrombosis by IBD and thrombosis status

	IBD		No IBD			
	Thrombosis	No thrombosis	Thrombosis	No thrombosis	1 vs. 2	1 vs. 3
	Group 1	Group 2	Group 3	Group 4	*p* -Value	
	Percent or mean (SE)					
No. of hospital admissions						
Actual	28,155	346,160	2,029,915	42,718,856		
Weighted	139,767	1,720,797	10,092,645	212,522,234		
Age (y)	57.48 (0.11)	50.44 (0.03)	65.01 (0.01)	47.88 (0.00)	<0.0001	<0.0001
< 18	0.75	3.80	0.65	16.50		
18–40	18.83	30.99	8.93	21.93		
> 40	80.42	65.22	90.42	61.56		
Race					<0.0001	<0.0001
Caucasian	81.54	79.68	73.2	65.4		
African American	10.98	10.65	16.53	14.90		
Asian	0.62	1.06	1.20	2.77		
Other	6.86	8.61	9.06	16.93		
Gender					0.0816	0.0002
Female	56.37	56.90	55.25	57.84		
Male	43.63	43.10	44.75	42.16		
Type IBD						
Regional enteritis	60.15	63.92	0	0	<0.0001	
Ulcerative colitis	40.14	36.45	0	0	<0.0001	
Thrombosis (%)						
Pulmonary embolism	6.46	0	10.50	0		<0.0001
Deep venous thrombosis	6.21	0	7.96	0		<0.0001
Arterial thrombosis/arterial embolism	0.03	0	0.09	0		0.0016
Intraabdominal thrombosis (%)						
Mesenteric vein thrombosis	7.45	0	3.94	0		<0.0001
Portal vein thrombosis	2.53	0	1.42	0		<0.0001
Other/splenic vein thrombosis	0.54	0	0.51	0		0.3792
Hepatic vein thrombosis (Budd–Chiari syndrome)	0.30	0	0.15	0		<0.0001
Other thromboembolism						
Cerebral artery embolism	2.16	0	5.48	0		<0.0001
Cerebral artery thrombosis	0.42	0	0.87	0		<0.0001
Retinal occlusion (artery or vein)	0.36	0	0.82	0		<0.0001
Intracranial venous sinus thrombosis	0.35	0	0.26	0		0.0021
Risk factors/comorbidities						
General surgery	53.73	51.87	45.61	50.45	<0.0001	<0.0001
Abdominal surgery	24.72	26.33	8.16	4.73	<0.0001	<0.0001
Ports/central venous catheters	13.19	7.65	8.72	3.88	<0.0001	<0.0001
Malignancy	13.10	9.18	18.62	9.88	<0.0001	<0.0001
Dehydration	12.43	13.21	7.75	6.24	0.0002	<0.0001
Malnutrition	8.87	5.79	4.92	2.34	<0.0001	<0.0001
Long-term steroid use	8.20	5.51	2.31	0.93	<0.0001	<0.0001
Orthopedic surgery	5.19	4.92	7.51	8.36	0.0403	<0.0001
Trauma	5.03	4.08	7.42	6.93	<0.0001	<0.0001
Medical conditions						
Hypertension	45.17	36.61	62.51	41.79	<0.0001	<0.0001
Smoking	26.65	26.09	26.43	19.93	0.0427	0.4201
Hyperlipidemia	21.50	17.77	33.59	22.62	<0.0001	<0.0001
Cardiac disease	16.61	12.73	26.24	17.76	<0.0001	<0.0001
Diabetes	14.95	12.14	22.82	16.32	<0.0001	<0.0001
Renal failure	13.55	8.90	18.05	10.83	<0.0001	<0.0001
Obesity	10.44	7.55	14.85	9.34	<0.0001	<0.0001
Cirrhosis	1.72	1.25	1.18	0.79	<0.0001	<0.0001
Liver disease	1.66	1.47	1.07	0.76	0.0118	<0.0001
Hepatitis C	1.32	1.49	1.69	1.61	0.0187	<0.0001
HIV	0.48	0.48	0.74	0.62	0.9626	<0.0001
Transfusion						
Blood or blood products	0.01	0.01	0.02	0.01	0.6889	0.4667
Platelets	1.28	0.70	1.20	0.67	<0.0001	0.2550
Coagulation factors	0.05	0.02	0.05	0.03	0.0153	0.7531
Charlson's comorbidity index	1.42 (0.01)	0.96 (0.00)	2.17 (0.00)	1.20 (0.00)	<0.0001	<0.0001
Length of Stay	7.75 (0.06)	5.46 (0.01)	6.72 (0.01)	4.48 (0.00)	<0.0001	<0.0001
Discharge Status (inpatient mortality)	3.76	1.49	4.47	1.78	<0.0001	<0.0001

Abbreviations: IBD, inflammatory bowel disease; SE, standard error.

Among venous thromboses, mesenteric vein thrombosis was the most common type. Patients with IBD were nearly twice as likely to have mesenteric vein thrombosis (7.45 vs. 3.94%, *p* < 0.0001) or portal vein thrombosis (2.53 vs. 1.42%, *p* < 0.0001), as patients without IBD. By contrast, pulmonary embolism (6.46 vs. 10.50%) and deep venous thrombosis (6.21 vs. 7.96%) were significantly lower in IBD than in those without IBD, each *p* < 0.0001. Among arterial thromboses, cerebral arterial embolism (2.16 vs. 5.48%, *p* < 0.0001) and cerebral arterial thrombosis (0.42 vs. 0.96%, *p* < 0.0001) were also lower in IBD patients ( Table 2 ).

### Univariate Analysis

Among patients with IBD, those with thrombosis were older ( *p* < 0.0001) and more likely to be Caucasian ( *p* < 0.0001) than those without thrombosis ( Table 2 ). Patients who developed thrombosis were more likely to have had a surgical procedure (53.73 vs. 51.87%), a port or central venous catheter (CVC; 13.19 vs. 7.65%), malignancy (13.10 vs. 9.18%), malnutrition (8.87 vs. 5.79%), received long-term steroids (8.20 vs. 5.51%), or suffered trauma (5.03 vs. 4.08%), all *p* < 0.0001, compared with patients without thrombosis. Patients with thrombosis were slightly more likely to have had orthopaedic surgical procedures (5.19 vs. 4.92%), *p* = 0.04, but less likely to be dehydrated (12.43 vs. 13.21%) or to have had abdominal surgery (24.72 vs. 26.33%), *p* < 0.0001 ( Table 2 ).

Among all thrombosis patients, comorbid conditions including cirrhosis and liver disease were significantly more common in IBD patients, while hypertension, hyperlipidemia, cardiac disease, diabetes, renal failure, obesity, hepatitis C, and HIV were significantly less common, as compared with non-IBD patients; all *p* < 0.0001. Smoking was not different between the groups, while markers of overall health, including the Charlson's score (1.42 vs. 2.17, *p* < 0.0001) and inpatient mortality (3.76 vs. 4.47%, *p* < 0.0001) were significantly less severe and length of stay (7.75 vs. 6.72 days, *p* < 0.0001) longer in IBD patients with thrombosis ( Table 2 ).

### Multivariable Logistic Regression

After adjusting for age and severity of illness with multivariable logistic regression, risk factors remaining significant for thrombosis in IBD patients included the presence of a port/central venous catheter (OR = 1.73, 95% CI: 1.66–1.80), long-term steroid use (OR = 1.61, 95% CI: 1.54–1.69), malnutrition (OR = 1.34, 95% CI: 1.27–1.40), and malignancy (OR = 1.13, 95% CI: 1.08–1.18; Table 3 ). By contrast, Asian race (OR = 0.61, 95% CI: 0.52–0.72) appeared to be protective against thrombosis. Several factors, while statistically significant, did not appear to be clinically strong risk or protective factors, including general surgery, renal failure, hyperlipidemia, and dehydration ( Table 3 ). Further multivariate analysis, after adjusting for potential confounders, that is, the variables in Table 3 , confirmed the difference in thrombosis rate between IBD and non-IBD patients ( Table 4 ), similar to the model in which only age was adjusted.

**Table 3:** Multivariable logistic regression odds ratios for thrombosis in IBD

Covariate	OR (95% CI)	*p* -Value
Port	1.73 (1.66–1.80)	<0.0001
Long-term steroid use	1.61 (1.54–1.69)	<0.0001
Malnutrition	1.34 (1.27–1.40)	<0.0001
Race Caucasian	Reference	
African American	1.14 (1.10–1.19)	<0.0001
Asian	0.61 (0.52–0.72)	<0.0001
Other	0.88 (0.84–0.93)	<0.0001
Malignancy	1.13 (1.08–1.18)	<0.0001
Charlson's comorbidity score	1.09 (1.08–1.10)	<0.0001
Age	1.02 (1.01–1.02)	<0.0001
Trauma	1.02 (0.96–1.08)	0.4993
Hypertension	1.01 (0.98–1.05)	0.3560
General surgery	0.96 (0.93–0.98)	0.0021
Renal failure	0.95 (0.90–0.99)	0.0285
Hyperlipidemia	0.94 (0.90–0.97)	0.0002
Dehydration	0.86 (0.83–0.90)	<0.0001

Abbreviations: CI, confidence interval; IBD, inflammatory bowel disease; OR, odds ratio.

**Table 4:** Multivariable logistic regression odds ratios for thrombosis in all patients

Covariate	OR (95% CI)	*p* -Value
IBD a	1.74 (1.71–1.76)	<0.0001
Port	2.05 (2.03–2.06)	<0.0001
Long-term steroid use	1.74 (1.72–1.76)	<0.0001
Malnutrition	1.28 (1.27–1.29)	<0.0001
Race Caucasian	Reference	
African American	1.19 (1.18–1.19)	<0.0001
Asian	0.50 (0.50–0.51)	<0.0001
Other	0.70 (0.70–0.71)	<0.0001
Malignancy	1.30 (1.29–1.31)	<0.0001
Hypertension	1.12 (1.12–1.13)	<0.0001
Charlson's comorbidity score	1.12 (1.11–1.12)	<0.0001
Age	1.02 (1.02–1.02)	<0.0001
Hyperlipidemia	1.01 (1.01–1.02)	<0.0001
Trauma	0.92 (0.91–0.92)	<0.0001
Dehydration	0.86 (0.85–0.86)	<0.0001
General surgery	0.80 (0.80–0.81)	<0.0001
Renal failure	0.79 (0.79–0.80)	<0.0001

Abbreviations: CI, confidence interval; IBD, inflammatory bowel disease; OR, odds ratio.

a Adjusted prevalence (95% CI): IBD 6.39% (range: 6.31–6.47%) versus no IBD 3.44% (range: 3.43–3.44%).

## Discussion

This cross-sectional study of discharges in the NIS cohort demonstrates that the prevalence of thrombosis is 1.7-fold greater in those with IBD as compared with those without IBD, consistent with previous studies. 1 2 3 9 10 11 The most common site was intra-abdominal, that is, mesenteric vein thrombosis, as previously reported. 16 IBD patients with thrombosis were younger, and, accordingly also had lower comorbidity score and lower in-hospital mortality. Thrombosis-specific risk factors, including steroid use, malnutrition, dehydration, port use, abdominal surgery, and general surgery, were all significantly more common in IBD than non-IBD thrombosis patients, consistent with previous studies. 10 16 17 18 19 20 21 Malnutrition was likely associated with other risks for VTE, including requirement for port/central venous catheter, hospitalization, and/or surgery. 19 Further, women with IBD, similar to those without IBD, were significantly more likely than men to develop thrombosis, as previously reported, consistent with thrombosis risk in pregnancy and in the postpartum period. 22 23

Asian ethnicity was found to be protective, consistent with general risk of thromboembolism in this group. 24 25 By contrast, typical risk factors for VTE, including hypertension, hyperlipidemia, cardiac disease, diabetes, renal failure, and obesity, were significantly lower in IBD with thrombosis, likely, in part, related to their lower age.

Whether any risk groups for thrombosis among those with IBD would benefit from anticoagulation prophylaxis remains unknown. Thromboprophylaxis is typically avoided in patients with IBD due to concerns regarding GI tract bleeding. 26 Despite this, anticoagulation prophylaxis has been shown to be safe in IBD patients, with no greater bleeding than in non-IBD patients. 18 26 27 Further, it has been recommended that anticoagulation should be extended beyond acute thrombosis and/or hospitalization, 18 28 29 especially in those with frequent flares and chronic steroid use. 17 18 30 31 Our findings indicate that thrombosis risk exists in other subsets of IBD patients, that is, those using ports and undergoing general and abdominal surgery. In fact, it has been shown that IBD patients receiving thromboprophylaxis within 24 hours of admission are half as likely to develop VTE. 18 However, more research is needed to identify those who might benefit from long-term anticoagulation.

The observation that thrombosis site differs between those with IBD and those without IBD is of interest. Specifically, those with IBD were nearly twice as likely to have mesenteric or portal vein thrombosis, but significantly less likely to have pulmonary embolism, deep venous thrombosis, cerebral arterial embolism, or cerebral arterial thrombosis than in those without IBD. This suggests the possibility that the pathophysiology of IBD may involve prothrombotic signaling or pathways that promote local thrombosis in the GI tract. Further mechanistic studies of IBD thrombosis might lead to a better understanding of thrombosis, in general, and potentially identify new targets for thrombosis prevention.

Why mortality was significantly lower in individuals with IBD, compared with those without IBD is unknown. It is possible, but not proven that lower IBD mortality could be related to the lower prevalence of African American ethnicity, a group with poorer access to care and poorer health outcomes; or the lower IBD mortality could be related to a lower prevalence of malignancy and associated shortened survival. However, these are not proven, and more research is needed to determine the causes for reduced mortality in IBD.

## Limitations

There are several limitations to this study. First, as the NIS is an inpatient database, there is potential bias toward a sicker population, as healthier patients who did not require admission are not included, introducing selection bias. Second, the NIS represents only 20% of the total inpatient population, so discharge-level-weights were used to determine a representative sample. Third, this sample is dependent on discharge diagnoses codes, which are limited by coding accuracy and potential misclassification bias, and further prevent tracking of the individual patient as the data are of discharge level rather than patient level. For the same reason, it is not possible to determine whether thrombosis at the patient level was provoked, symptomatic, or whether thromboprophylaxis was given. However, previous studies examining hospital discharge data have indicated sufficient accuracy for use in research studies. 32 33 Fourth, as this was a retrospective study, it is subject to bias and cannot be used to determine causality. Fifth, the NIS database does not contain laboratory values or drug treatment information, and thus it cannot be used to assess the relation between thrombosis risk and severity of IBD, duration of steroids or port use, or use of thromboprophylaxis, nor is it possible to adjust results for IBD duration or disease activity. A significant strength of the NIS is its size, this sample offers a large patient pool to assess an uncommon disease like IBD.

## Conclusion

In conclusion, this study confirms that venous thrombosis is significantly increased among IBD patients. Recognition of those at high risk may help to identify potential patients for thromboprophylaxis safety and efficacy studies. Future trials will be critical to develop evidence for the optimal management of these patients.
